# Awareness and use of HIV pre-exposure prophylaxis and factors associated with awareness among MSM in Beijing, China

**DOI:** 10.1038/s41598-023-27485-8

**Published:** 2023-01-11

**Authors:** Yanming Sun, Hongyan Lu, Jingrong Ye, Dongmin Li, Guiying Li

**Affiliations:** 1 Institute for HIV/AIDS and STD Prevention and Control, Beijing Center for Disease Prevention and Control (CDC), No. 16, Hepingli Middle Street, Dong Cheng District, Beijing, 100013 China; 2 National Center for AIDS/STD Control and Prevention (NCAIDS), China CDC, Beijing, China

**Keywords:** HIV infections, Epidemiology

## Abstract

Human immunodeficiency virus (HIV) sexual transmission among men who have sex with men (MSM) has increased markedly in Beijing, China in the past decade. Pre-exposure prophylaxis (PrEP) is a highly efficacious biomedical prevention strategy that remarkably reduces HIV-transmission risk. This study examined PrEP awareness among MSM and the factors influencing it. From April to July 2021, respondent-driven sampling was used to conduct a cross-sectional survey among MSM in Beijing, China. Demographic, behavior, and awareness data regarding PrEP were collected. The factors influencing PrEP awareness were assessed using univariate and multivariable logistic regression. In total, 608 eligible responders were included in the study. Among the respondents, 27.9% had PrEP awareness, 3.3% had taken PrEP, and 57.9% expressed interest in receiving PrEP, if required. Greater odds of PrEP awareness were associated with higher education level (adjusted odds ratio [aOR] 3.525, 95% confidence interval [CI] 2.013–6.173, P < 0.0001), greater HIV-related knowledge (aOR 3.605, 95% CI 2.229–5.829, P < 0.0001), HIV testing (aOR 2.647, 95% CI 1.463–4.788, P = 0.0013), and sexually transmitted infections (aOR 2.064, 95% CI 1.189–3.584, P = 0.0101). Lower odds of PrEP awareness were associated with higher stigma score (aOR 0.729, 95% CI 0.591–0.897, P = 0.0029). The findings indicate sub-optimal awareness and low utilization of PrEP in Beijing and highlight PrEP inequities among MSM with stigma. Strengthening the training of peer educators in disseminating PrEP knowledge and reducing stigma are critical for improving PrEP awareness.

## Introduction

Men who have sex with men (MSM) in China are at a risk of HIV infection. HIV prevalence among MSM has increased from 0% in 2001 to 22.91% in 2018^1^. Sexual transmission by MSM is the most commonly reported route of HIV transmission in Beijing. This group is disproportionately affected by HIV and accounts for 77.97% of newly diagnosed cases^2^. Accordingly, MSM is considered a critical group to access and use antiretroviral pre-exposure prophylaxis (PrEP). PrEP, a biomedical HIV prevention method, reduces the risk of sexual acquisition by more than 90% when taken with high adherence^3^.

Furthermore, PrEP is cost-effective based on modeling studies conducted in Germany, UK, and the Netherlands^4–6^. The World Health Organization has recommended the use of PreP in 2014 after the first PrEP studies have been published^7^. PrEP is an important biomedical tool for HIV prevention, and more than 50 countries and regions have approved its use^8^. In China, considering the price, as well as the adherence and potential risk of PrEP use, its actual acceptance is limited. PrEP only became available for uninfected people in 2020^9^. National guidelines based on oral emtricitabine/tenofovir were issued in 2021^10^. Individuals who require PrEP needs to be aware of its existence. However, related studies in China are limited. The most recent study was conducted in southern China with awareness of 43.1% in 2017^11^ and 52.7% in 2019^12^. Considering that PrEP knowledge is a prerequisite (although not sufficient) for its acceptability, the current study aimed to elucidate the behavioral and demographic parameters that are correlated with PrEP. These parameters toned to be identified to provide population-specific information. We aimed to study the factors associated with PrEP awareness among MSM. The results of our analysis will help policymakers conceptualize better strategies to increase awareness and use of PrEP among MSM.

## Methods

### Study design and participants

Cross-sectional surveys were conducted from April to July 2021 in Beijing. The target population included MSM aged 18 years or above who reported oral or anal sex with at least one male sex partner in the half past year. Individuals with mental disabilities were excluded from the survey.

### Sampling and recruitment

The survey was administered using the respondent-driven sampling (RDS) method^13,14^. RDS is a peer-referral sampling methodology for the estimation of the characteristics of underserved groups that cannot be randomly sampled. It is often implemented to identify hidden populations at risk for HIV.

From April to July 2021, nine MSM with different demographic characteristics were selected as survey seeds, and three recruitment cards were issued to each participant. They subsequently selected three MSM from their friends to participate in the survey. The seeds had to meet the following inclusion criteria^15^: (a) willing to recruit other MSM participants, (b) have a wide range of contacts in the MSM community, and (c) have a selection of seeds from across all types of demography. Seeds were selected according to age, marital status, education, sexual orientation, and HIV testing, and they exhibited various types of these five aspects. Each participant had a recruitment card to participate in the survey. After completing the survey, each participant received three recruitment cards. Recruitment was continued until the sample reached stability.

**Figure 1:**
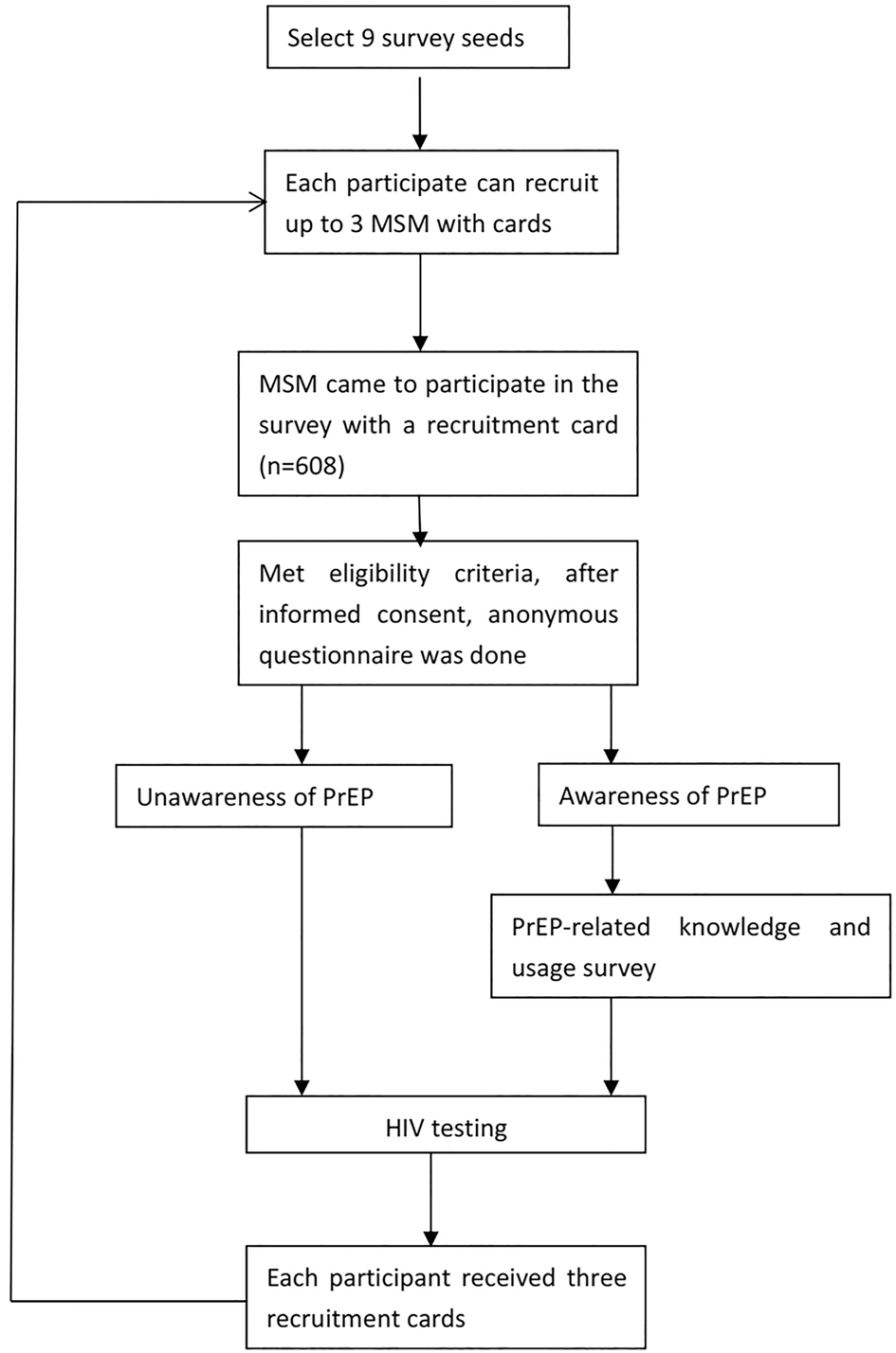
Flow chart of eligibility and enrollment.

After obtaining written informed consent from the participants, each subject underwent an anonymous interview for data gathering. The participants were interviewed by skilled interviewers in a private room at the voluntary counseling and testing clinic of the Beijing Centers for Disease Control and Prevention (CDC). A cash reward of 50 Chinese Yuan (CNY; approximately 7–8 United States dollars) was awarded for participation in the questionnaire survey. Upon recruiting three MSM, each participant received a reward of 100 CNY. As part of the national HIV sentinel surveillance program^16,17^, this study was approved by the Institutional Review Board of the National Center for AIDS/STD Control and Prevention, China CDC, (IRB0000276 and FWA00002958). All methods were performed in accordance with relevant guidelines and regulations (Fig. 1).

### Sample size

Recruitment was continued until the overall sample reached stability or ‘‘equilibrium’’ and the projected sample sizes were met^18^. Stability was assessed by monitoring the progress of key variables throughout the recruitment process and is considered to be achieved when the proportion of these indicators does not substantially change with the subsequent waves of participants (the cumulative sample proportion at each wave does not change by more than 1.0%)^14,19^. Stability in age, education, marital status, sexual orientation, and HIV test in the past year were tracked. During the investigation, the sample equilibrium test of the main indicators was carried out continuously.

### Measurements

The research team developed a structured questionnaire. Except for PrEP-related questions, a survey questionnaire that had been previously used in annual MSM surveys^20^ in Beijing for more than 10 years was employed. PrEP-related issues were added by the research team in 2021 and pre-tested in 12 young adults (not included in the study).

The questionnaire was used to collect data, including demographic characteristics (e.g., age, education, marital status, and residency), frequency of condom usage during sexual contact, and previous HIV testing. PrEP awareness among MSM was assessed by asking about their awareness in PrEP [‘Have you ever heard of PrEP and its intended purpose? Please explain’]. In the evaluation of the willingness of MSM to use PrEP, only people with PrEP awareness and did not take it were asked about their willingness to use it [‘Would you like to use PrEP?’]. The willingness to use PrEP was asked based on a 3-point Likert scale (1 = definitely will not, 2 = unsure/probably will, 3 = definitely will). Participants were classified into the “willing to use PrEP” group when they endorsed responses 2 or 3. Participants who did not know about PrEP were not asked any further questions.

Participants who knew about PrEP but had not used it were asked about their reasons for declining its usage. Eight knowledge questions were included in this survey, and five of which were extracted from the United Nations General Assembly Special Session^21^. The statements were as follows: (1) people can protect themselves from contracting HIV by having sex with only one faithful, uninfected partner; (2) people can protect themselves from contracting HIV by using condoms; (3) a healthy-looking person can have HIV; (4) a person can acquire HIV from mosquito bites; and (5) a person can acquire HIV by sharing a meal with someone who is infected. The remaining three questions regarded the HIV transmission route: (1) a person can acquire HIV from receiving HIV-infected blood, (2) a person can acquire HIV by sharing needles with an infected individual, and (3) children born to HIV-infected women may contract HIV. All correct answers were considered as “good knowledge of HIV” and otherwise considered as “poor knowledge of HIV”. The participants’ degree of stigmatization of homosexuality was determined by asking the respondents three questions as follows^22^: “If a relative is part of the MSM population, will others feel ashamed?”; “Would you hide your MSM identity to avoid discrimination?”; and “Are you ashamed after having sex with other men?” Participants rated each item on a 3-point scale (0 = never/strongly disagree, 1 = sometimes/partially agree, 2 = very often/strongly agree). The final stigma score was the sum of the three responses.

### Statistical analysis

The questionnaire data were entered and cleaned using EpiData software (version 3.1, Epidata Association, Odense, Denmark). All results in this analysis were reported in crude by using SPSS software (Version 19.0, SPSS, Inc.,Chicago, IL,USA). For the data collected by RDS, weighting was used to adjust for respondents’ social network size^23^ (i.e., the larger a social network, the greater the likelihood that someone might be recruited by other participants in his social network) and recruitment patterns. The size of the social network of a MSM was measured as the number of other MSM they knew by name or nickname, by face, and who were 18 years or older, lived in Beijing, and whom they could reach. RDS adjustment was calculated using RDS Analyst Software (version 0.57) based on the differences in the social network sizes of participants^24^. Individualized weights of HIV (outcome variable) were calculated using RDS Analyst Software, and then exported to SAS (version 9.2). Factors associated with PrEP awareness were analyzed using survey logistic regression models by presenting odds ratio (OR) and adjusted OR (aOR). Independent variables with P < 0.10 in univariate analysis were included in multivariable regressions. Statistical significance was set at P < 0.05.

## Results

### Population characteristics

**Figure 2:**
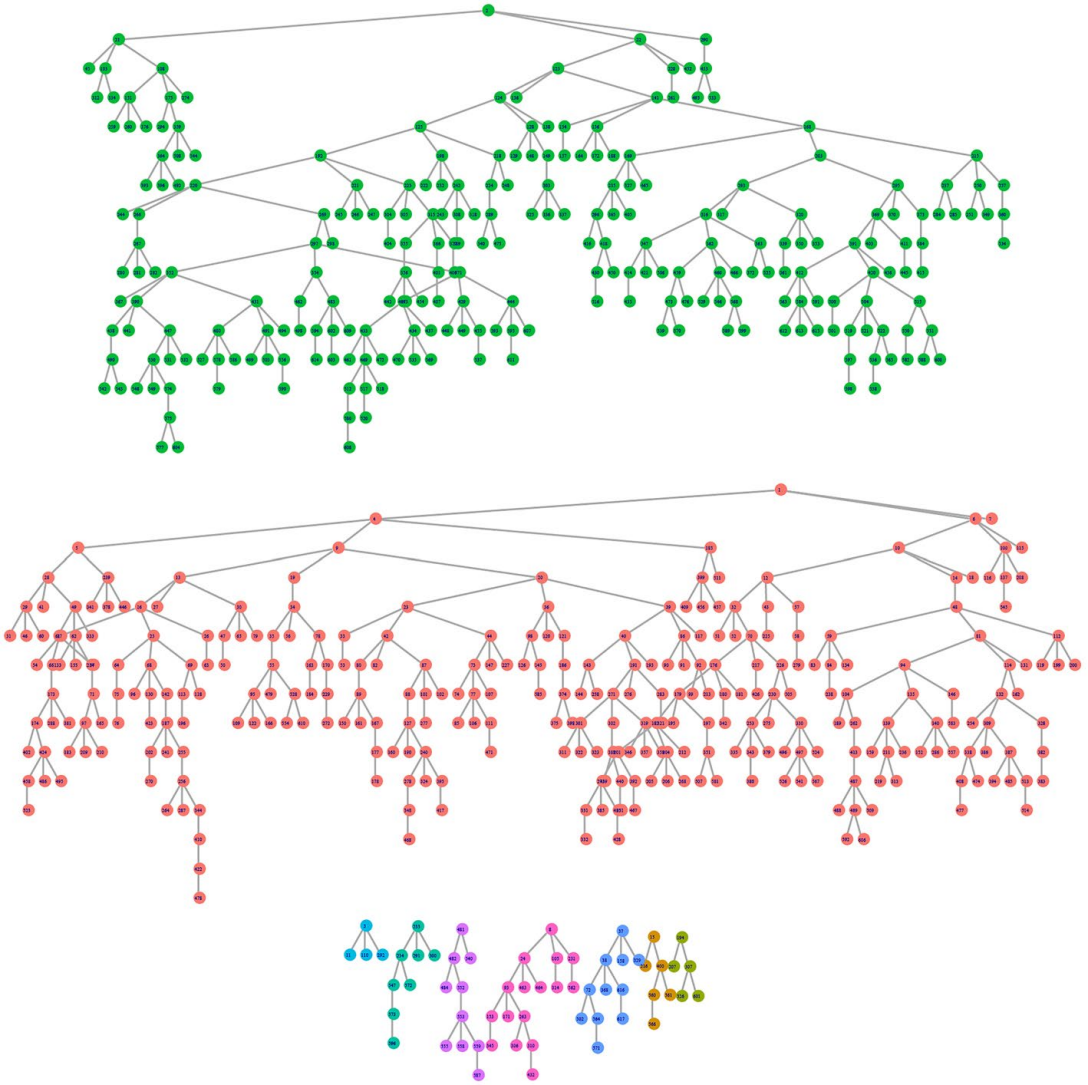
Recruitment trees of MSM in 2021. Each point represents a participant, the recruitment order is from top to bottom, and the short line represents the recruitment relationship. Dots of the same color indicate those recruited by the same seed.

**Figure 3:**
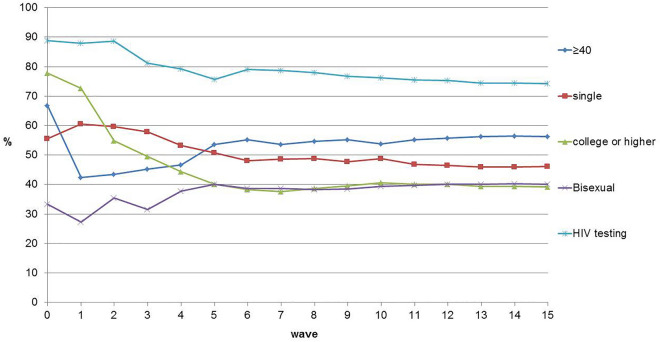
Sample equilibrium by age, martial status, education, sexual orientation and HIV testing.

A total of 608 MSM were enrolled in this study. The recruitment trees used in the study are shown in Fig. 2. The main demographic indicators of age, marriage, education level, sexual orientation, and HIV testing in the past 12 months were balanced at waves 12, 12, 11, 7, and 10 (Fig. 3). The sample is then considered to be in equilibrium.

The average age of MSM was 41.6 years (SD ± 11.0). Table 1 demonstrates the characteristics of demographics, condom use, HIV testing history, and HIV knowledge among the recruited MSM. Among the respondents, 37.9% had a college degree or higher. Homosexual orientation was observed in 58.3% of participants. Consistent condom use in sexual intercourse during the preceding 6 months was observed in 53.0% of the study population, and the self-reported history of contracting sexually transmitted infections (STIs) was 16.6%. Among the surveyed MSM, 33.2% were involved in sexualized drugs used in the past 6 months, and 70.9% were tested for HIV within the past 12 months. During the study, 81.7% of MSM received peer-educator support. HIV knowledge was identified in 49.0% of the MSM. The average stigma score was 3.7 (SD ± 1.2).

**Table 1:** Demographic and behavioral characteristics of MSM recruited in 2021 in Beijing, China (N = 608).

Categorical variables	Crude		RDS-adjustment	
	n	%	%	95%CI
**Age (years)**				
< 25	27	4.4	4.8	2.9–6.7
25–39	239	39.3	37.0	32.3–41.7
≥ 40	342	56.3	58.2	53.1–63.2
**Marital status**				
Single	281	46.2	44.1	38.5–49.7
Married/cohabiting	238	39.1	41.1	35.9–46.2
Divorced/widowed	89	14.6	14.9	11.5–18.3
**Education**				
Middle school or lower	370	60.9	62.1	56.2–68.0
College or higher	238	39.1	37.9	32.0–43.9
**Time in Beijing (years)**				
≥ 2	523	86.0	85.9	82.5–89.3
< 2	85	14.0	14.1	10.7–17.5
**Monthly income (CNY)**				
< 5000	279	45.9	47.5	42.6–52.4
5000–10,000	218	35.9	35.2	30.9–39.4
≥ 10,000	111	18.3	17.4	13.8–21.0
**Sexual orientation**				
Bisexual	243	40.0	41.1	36.3–45.9
Homosexual	362	59.5	58.3	53.5–63.1
Heterosexuality	2	0.3	0.4	0–0.7
Asexual	1	0.2	0.2	0–0.6
**Main means of meeting partners**				
Face-to-face directly	224	36.8	37.8	33.4–42.2
Internet	384	63.2	62.2	57.8–66.6
**Self-assessed risk of HIV infection**				
Low	472	77.6	76.8	72.9–80.6
High	136	22.4	23.3	19.4–27.1
Awareness of PrEP	185	30.4	27.9	23.7–32.2
Previous PrEP use	21	3.5	3.3	1.3–5.2
**No of MSM partners in the preceding 6 months**				
1	168	27.6	28.7	24.4–33.0
2–9	352	57.9	58.0	53.8–62.2
≥ 10	88	14.5	13.3	10.2–16.3
**Condom use in MSM anal sex during the preceding 6 months**^**a**^				
Consistent	268	54.5	53.0	48.1–58.0
Inconsistent	224	45.5	47.0	42.1–52.0
Sexualized drug used during the preceding 6 months	207	34.0	33.2	28.8–37.6
STIs history	113	18.6	16.6	13.6–19.5
Peer education in preceding year	501	82.4	81.7	78.1–85.3
HIV testing in the preceding 12 months	451	74.2	70.9	65.9–76.0
Good knowledge of HIV	308	50.7	49.0	44.2–53.8

Continuous variable	Mean	SD	Mean	95%CI
Stigma^b^	3.7	1.2	3.7	3.6–3.8

^a^492 MSM had MSM anal sex during the preceding 6 months.

^b^measured on a 7-point scale from 0 to 6, 6 = extremely stigma.

### Awareness, knowledge, and use of PrEP

Among the participants, 27.9% had heard of PrEP before participating in the study. A few respondents (3.3%) had taken PrEP. Table 2 shows the knowledge of PrEP among MSM. A total of 57.9% indicated intention to use PrEP in the future, if required. Participants who had heard PrEP but never taken it were asked about the reasons, and among the participants who responded to these items, 35.2% agreed that they would not take PrEP, because they preferred using condoms as protection for HIV, 28.6% had a fluke mentality, 15.1% are concerned about side effects, and 9.3% felt that they could not afford it.

**Table 2:** Knowledge of PrEP among MSM recruited in 2021 in Beijing, China (n = 185) (Calculated for those who knew about PrEP).

	Crude		RDS-adjustment	
	n	%	%	95%CI
**Where to purchase PrEP**				
Know	150	81.1	79.8	72.0–87.7
Unknown	35	18.9	20.1	12.3–28.0
**Reasons for not taking**^**a**^				
Expensive	17	10.4	9.3	4.1–14.6
Side effects	24	14.6	15.1	8.7–21.5
Stigma	4	2.4	2.5	0–5.3
Consistent condom use	59	36.0	35.2	25.3–45.1
Fluke mentality	44	26.8	28.6	18.4–38.8
Unknowing how to access	4	2.4	2.6	0–5.3
Previously HIV positive	12	7.3	6.6	1.3–12.0
**Likelihood of using PrEP**^**b**^				
Yes	98	57.6	57.9	50.4–65.5
No	72	42.4	42.1	34.5–49.6

^a^Calculated for those who had not used PrEP, n = 164.

^b^Calculated for those who were HIV negative, n = 170.

### Factors related to PrEP awareness

Univariate and multivariable analyses were performed to explore possible factors associated with PrEP awareness (Table 3). Multivariable regression results showed that MSM were three more likely to be aware of PrEP if they had a college or higher degree (aOR 3.525, 95% CI 2.013–6.173, P < 0.0001). MSM with good HIV knowledge were more likely to hear about PrEP (aOR 3.605, 95% CI 2.229–5.829, p < 0.0001). Other factors, including the use of sexualized drugs in the past 6 months (OR 2.059, 95% CI 1.276–3.322, P = 0.0031), HIV testing in the past 12 months (aOR 2.647, 95% CI 1.463–4.788, p = 0.0013), and STI infection (aOR 2.064, 95% CI 1.189–3.584, P = 0.0101) were associated with increased PrEP awareness. MSM who had higher stigma score (aOR 0.729, 95% CI 0.591–0.897, P = 0.0029) were less likely to hear about PrEP.

**Table 3:** Factors associated with the PrEP awareness among MSM recruited in 2021 in Beijing, China.

Categorical variables	Awareness		Unawareness		Univariate analysis		Multivariate analysis	
	N	%	N	%	OR (95%CI)	P	aOR (95%CI)	P
**Age (years)**								
≥ 40	67	19.6	275	80.4	1		1	
25–39	108	45.2	131	54.8	3.832 (2.583–5.685)	< 0.0001	1.450 (0.803–2.619)	0.2182
< 25	10	37.0	17	63.0	2.372 (0.971–5.796)	0.0581	1.180 (0.348–3.996)	0.7905
**Marital status**								
Single	124	44.1	157	55.9	1		1	
Married/cohabitating	39	16.4	199	83.6	0.251 (0.159–0.397)	< 0.0001	0.555 (0.286–1.076)	0.0814
Divorced/widowed	22	24.7	67	75.3	0.376 (0.212–0.664)	0.0008	0.695 (0.325–1.486)	0.3478
**Time in Beijing (years)**								
< 2	17	20.0	68	80.0	1		1	
≥ 2	168	32.1	355	67.9	1.771 (0.973–3.226)	0.0616	1.249 (0.622–2.507)	0.5311
**Education**								
Middle school or lower	58	15.7	312	84.3	1		1	
College or higher	127	53.4	111	46.6	6.290 (4.165–9.500)	< 0.0001	3.525 (2.013–6.173)	< 0.0001
**Monthly income (CNY)**								
< 5000	47	16.8	232	83.2	1		1	
5000–10,000	75	34.4	143	65.6	2.700 (1.723–4.231)	< 0.0001	1.117 (0.620–2.014)	0.7125
≥ 10,000	63	56.8	48	43.2	7.002 (4.123–11.889)	< 0.0001	1.494 (0.718–3.108)	0.2825
**Sexual orientation**^**a**^								
Bisexual	56	23.0	187	77.0	1		1	
Homosexual	128	35.4	234	64.6	1.856 (1.249–2.758)	0.0022	0.687 (0.408–1.155)	0.1562
**Main means of meeting partners**								
Face-to-face directly	55	24.6	169	75.4	1		1	
Internet	130	33.9	254	66.1	1.492 (1.003–2.220)	0.0483	0.888 (0.528–1.492)	0.6529
**Self-assessed risk of HIV infection**								
Low	144	30.5	328	69.5	1			
High	41	30.1	95	69.9	0.942 (0.603–1.472)	0.7940	/	
**Knowledge of HIV**								
Poor	46	15.3	254	84.7	1		1	
Good	139	45.1	169	54.9	4.700 (3.115–7.091)	< 0.0001	3.605 (2.229–5.829)	< 0.0001
**No of MSM partners in the preceding 6 months**								
1	41	24.4	127	75.6	1		1	
2–9	114	32.4	238	67.6	1.665 (1.061–2.612)	0.0266	1.245 (0.705–2.198)	0.4502
≥ 10	30	34.1	58	65.9	1.797 (0.989–3.265)	0.0542	0.897 (0.407–1.973)	0.7861
**Condom use in MSM anal sex during the preceding 6 months**^**b**^								
Consistent	87	32.5	181	67.5	1			
Inconsistent	74	33.0	150	67.0	0.964 (0.640–1.454)	0.8628	/	
**Sexualized drug used during the preceding 6 months**								
No	90	22.4	311	77.6	1		1	
Yes	95	45.9	112	54.1	3.031 (2.060–4.460)	< 0.0001	2.059 (1.276–3.322)	0.0031
**HIV testing in the preceding 12 months**								
No	26	16.6	131	83.4	1		1	
Yes	159	35.3	292	64.7	3.278 (1.997–5.380)	< 0.0001	2.647 (1.463–4.788)	0.0013
**STI history**								
No	140	28.2	356	71.8	1		1	
Yes	45	40.2	67	59.8	1.935 (1.221–3.069)	0.0050	2.064 (1.189–3.584)	0.0101
**Peer education in preceding 12 months**								
No	29	27.1	78	72.9	1			
Yes	156	31.1	345	68.9	1.456 (0.877–2.419)	0.1462	/	

Continuous variable	Awareness		Unawareness		Univariate Analysis		Multivariate Analysis	
	Mean	SD	Mean	SD	OR (95%CI)	P	aOR (95%CI)	P
Stigma score^c^	3.3	1.3	3.8	1.0	0.674 (0.573–0.792)	< 0.0001	0.729 (0.591–0.897)	0.0029

^a^ Sexual orientation removes heterosexuality and nonsexuality.

^b^ 492 MSM has MSM anal sex during the past six months.

^c^ measured on a 7-point scale from 0 to 6, 6 = extremely stigma.

## Discussion

This study was the first to investigate the awareness and use of PrEP among MSM in China by using RDS method. The results showed that the awareness and willingness on PrEP use were low. MSM with high education level, using sexualized drug, and undergoing HIV testing had a high awareness rate of PrEP, high MSM stigma would reduce the awareness rate. Peer education and MSM-related websites did not affect the promotion of PrEP.

RDS is a long-chain peer referral recruitment method and is a quasi-probability sampling method that allows population-based inferences through statistical adjustments^25^. A review^26^ of more than 120 RDS studies conducted worldwide found that RDS is an effective technique when designed and implemented appropriately for the sampling of most-at-risk populations for HIV biological and behavioral surveys. Samples generally reached equilibrium at approximately 10 waves^27^. The main indicators in this study reached equilibrium at waves 7–12. The sample represents MSM in Beijing to a certain extent.

### Awareness and willingness uptake of PrEP

In the present study, 27.9% of MSM had heard about PrEP. This awareness is better than those from surveys conducted in previous years in China (22.4% in 2017^28^, 22%^29^ and 11.2%^30^ in 2010), but it is lower than that in a recent research (52.7% in 2019^12^). Awareness on PrEP use also varies widely by geographical region; the rate of PrEP awareness among the present MSM was higher than that reported in Myanmar in 2014 (5%)^31^ and lower than the reported values in US in 2018 (54.8%)^32^ and Brazil and Malaysia in 2016 (61.3%^33^ and 44%^34^). This finding was obtained possibly because if PrEP is promoted for a long time^28–34^ and the HIV infection rate is high^12^, the promotion of PrEP will be accelerated. In addition, the survey carried out in the unit promoting PrEP will also result in increased awareness^12^. Therefore, the results of a study in one region or one study might not be generalizable worldwide.

Notably, the percentage of MSM who reported PrEP use was extremely low. The 3.3% uptake rate in the present study was similar to that in study of MSM in US in 2018 (2.5%)^32^. MSM who had previously heard of PrEP were much more likely to accept PrEP in the future^12,28^. However, in the present study, a brief introduction to PrEP did not effectively increase the willingness to use. Only 57.9% of MSM who had heard about PrEP indicated intent to use it in the future. This finding is similar to that in other studies in China (e.g. 64.0%^30^, 67.8%^12^, 71.3%^35^) and among YMSM in America (55.3%)^36^ and Lebanon (53.5%)^37^.

Similar to other studies^38–40^, the most common barrier to PrEP utilization is the belief that condom use is a more feasible form of HIV prevention than PrEP. In addition, although oral Tenofovir-based PrEP regimens are effective and safe for MSM^41–44^, 15.1% of MSM in the present sample were concerned about PrEP’s side effects. Even 28.6% believe that they are safe from HIV infection during unprotected sex. Therefore, the detailed effectiveness and safety profile of PrEP should be prioritized for PrEP initiation to maximize informed decision-making among potential users^45^. Therefore, physicians need to build awareness among at-risk populations who may benefit from PrEP and strengthen their knowledge training about PrEP.

### Factors associated with awareness of PrEP

#### Demographic characteristic

Consistent with previous studies, higher education levels were the associated with PrEP awareness among MSM^46–49^. This finding may be related to the unique national conditions and policies regarding the promotion of PrEP or a strong understanding and acceptance of new modalities.

#### High-risk sexual behavior

The influence of different risky behaviors on PrEP awareness was different. MSM who took sexualized drug had better awareness than those who did not. However, the number of sexual partners and anal condom use did not affect the awareness of PrEP.

Sexualized drug use refers to the use of any psychoactive substance before or during sexual intercourse^50^. Psychoactive substances adversely affect users’ capacity to perceive and respond to risks during sexual encounters, leading to high-risk sexual practices^51^ and infection with HIV and other STIs^52^. In the present study, one-third of MSM took sexualized drug within six months, which is higher than the reported value in Hong Kong 2018 (14.1%)^53^ and lower than those in UK (41%)^54^ and Australia (54%)^55^. Mathematical models suggested that achieving 75% PrEP coverage among high-risk HIV-negative MSM in China would prevent 25.7% of new HIV infections among all MSM^56^. MSM with experience on recent sexualized drugs may be a priority group for future PrEP implementation. In the present study, although MSM using sexualized drug had a higher awareness of PrEP, approximately 60% of the MSM who took sexualized drug remained unaware of PrEP, highlighting that effective strategies to promote PrEP are needed for this group of MSM in China.

Consistent with other studies^46,49,57,58^, the awareness of PrEP in the present study was not associated with the number of MSM partners or condom use in MSM anal sex. This finding was obtained possibly because the popularity of PrEP is insufficient (less than half of MSM had heard of PrEP), especially among MSM with risky sexual behaviors.

### MSM and PrEP stigma

In the present study, MSM had moderate MSM stigma. Low stigma levels related to sexual orientation are also related to increased PrEP awareness. Stigma can be perceived as a threat to social identity among MSM. The negative effect of stigma on PrEP awareness may be explained by social isolation and the lack of supportive networks. This finding corroborates the findings of other studies^59–61^. MSM stigma is also inextricably linked to PrEP stigma^62,63^, because MSM are currently the main recipients of PrEP for HIV infection prevention.

Although PrEP stigma was not serious in the present study, it remarkably affected the use of PrEP in previous studies. Rather than being viewed as merely an alternative and equally acceptable-prevention strategy, PrEP is considered a less honorable prevention choice^64^. This finding was obtained possibly because of the high degree of stigmatization of homosexuality^65^ and PrEP use being seen as a marker of promiscuity^66^. Considering the use of PrEP, safe behavioral constraints are lifted, and they are free to engage in sexual activity and condomless sex without fear of infection. Although the participants in this study stated that their PrEP stigma was low, this finding may not reflect the actual situation possibly because of PrEP stigma, in which many people said that they only rely on condoms 100% and do not need to take PrEP.

#### HIV testing and intervention services

PrEP awareness among MSM who have been tested is 2.647 times than those in untested people. Survey participants who underwent HIV-testing in the previous year had received a certain degree of HIV-related counseling, which should include education regarding prevention. Consistent with previous studies, previous HIV testing could be a marker of higher awareness for HIV risky behavior or reflect previous counselling^67^. These findings also supported the use of HIV testing as an entry point for biomedical and behavioral HIV prevention, including PrEP^58^. Previous STIs can also increase PrEP awareness, indicating that PrEP knowledge is received during healthcare consultation. However, approximately 60% of MSM who had been tested for STIs remained unaware of PrEP, highlighting a missed opportunity for targeted counseling.

Well-trained peer educators are critical in terms of delivering accurate knowledge of HIV^68^ and promoting harm-reduction interventions (e.g., condom use, encouraging regular HIV testing, and psychological support) through outreach activities^68–70^ and decreasing stigma-related HIV^71^. In the present study, the majority of MSM received peer education in the past 12 months. However, this condition was not related to PrEP awareness. However, peer educators may play an important role in future PrEP implementation^72,73^, because they are the main providers of HIV testing services for MSM in Beijing. Therefore, considering that PrEP is a relatively novel HIV prevention method, health departments should target counselling providers and peer educators for training on PrEP education and referral resources. This may help improve awareness and access to PrEP for at-risk MSM.

In the present study, online promotion did not improve PrEP awareness. MSM constitute a hidden population, and it is difficult to disseminate health-related information through the mass media. MSM-related and institutional websites have become the main channels of awareness. Since 2019, the China CDC has implemented programs and mobilized funding to support the Beijing Health Department and non-governmental organizations in PrEP implementation. They also provided extensive PrEP information online, which can be an effective resource for education/outreach programs. MSM who use the Internet as their main way of meeting sexual partners may also come across with MSM-related or institutional websites containing information about preventive measures^74^. However, no such connection was observed in the present study possibly because of insufficient online PrEP promotion.

## Limitations

Our study has certain limitations. First, considering the cross-sectional design, only the association was evaluated and not the causality of the risk factors for PrEP awareness. Second, although RDS was used to improve sample representation, some biases were incurred based on how the “seeds” were selected and how MSM recruited. For example, the monetary incentives for participation in RDS may have had a much stronger appeal to the lower socio-economic status MSM than higher SES MSM^75^. Lastly, behavioral information relied on self-reporting, which might have been influenced by recall and social desirability biases.

## Conclusions

MSM at high risk of HIV infection have moderate awareness of PrEP and low willingness to use PrEP. PrEP requires more promotion that focuses on at-risk MSM who have lower education, risky sexual behavior, and use sexualized drugs. Additionally, efforts should be made to reduce PrEP-related stigma and strengthen the training of healthcare providers and peer educators to improve the dissemination of PrEP knowledge.

## Data Availability

The datasets used and analyzed during the current study are available from the corresponding author on reasonable request.
